# Chinese and Korean Consumers’ Preferences for Oolong and Black Oolong Teas

**DOI:** 10.3390/foods14193327

**Published:** 2025-09-25

**Authors:** Baihan Su, Jeehyun Lee

**Affiliations:** Department of Food Science and Nutrition & Kimchi Research Institute, College of Human Ecology, Pusan National University, Busan 46241, Republic of Korea; jmssbh@pusan.ac.kr

**Keywords:** consumption habits, cross-cultural comparison, CATA-Penalty

## Abstract

Both China and Korea have significant markets for tea; however, both the availability and preference for oolong and black oolong tea vary across the two markets. Although this difference has been highlighted in previous studies, the group differences in the two markets based on preferences and sensory perceptions have not been assessed. Therefore, this study aimed to investigate the overall oolong and black oolong tea preferences based on specific characteristics of the tea samples and the sensory perceptions of young Chinese and Korean consumers residing in South Korea. Twelve tea samples were selected for on-site evaluations. A Check-All-That-Apply (CATA) method with 20 sensory characteristics was used to identify the sensory characteristics perceived, supplemented by intensity ratings for key attributes. The CATA-Penalty analysis revealed sensory characteristics that significantly impacted overall liking. The results indicated that while Chinese and South Korean consumers exhibited some differences in overall preference trends, they shared similar preferences within specific tea categories. This reflects differences in preferences between Chinese and South Korean consumers for oolong and black oolong tea beverages and the possible impact of different cultural backgrounds on consumption habits. These findings provide insights into cross-cultural sensory preferences and the impact of the cultural background on tea beverage perception.

## 1. Introduction

Tea is classified according to the degree of fermentation into unfermented (green tea), semifermented (oolong tea), and fermented (black tea). Oolong tea, a semifermented traditional Chinese tea, has garnered global popularity because of its distinctive characteristics [1]. In China, tea is classified into six types: green, white, yellow, oolong, black, and dark tea [2]. Oolong tea, also known as Qing tea, is primarily produced in Fujian, Guangdong, and Taiwan, with Fujian Anxi County being the largest tea producer. Key varieties include Tieguanyin, Phoenix Narcissus, Oriental Beauty, and Red Oolong [3]. Oolong tea production involves picking, withering, shaking, frying, rolling, and roasting, and traces its evolution from Song Dynasty tribute teas around 1725. Black oolong tea, a further processed type, has recently emerged. Black oolong tea is a unique variant of traditional oolong tea that undergoes prolonged oxidation and roasting, giving it a brilliant color and mellower flavor [1]. Compared with black and green teas, oolong tea has fewer production areas and a lower output. In 2022, oolong tea production was expected to exceed 310,000 tons, accounting for 13% of China’s total tea production [4]. The modern trend of canned oolong tea beverages began in the 1980s when Ito En Ltd. (Tokyo, Japan) introduced Japan’s first canned oolong tea [5]. The shift from traditional hot brewing to room-temperature or chilled beverages, attributed to urban lifestyle changes, has popularized PET bottles owing to their portability and ease of storage [6]. This shift has led to the diversification and differentiation of tea beverage flavors. Despite the variety of black and green tea beverages, oolong tea beverages remain rare [7]. Both China and South Korea have significant tea beverage markets; however, differences in food cultures and lifestyles may lead to varying consumer preferences and consumption habits. Chinese consumers tend to focus on the aroma and appreciate the natural flavor and potential health benefits of tea consumption, whereas Korean consumers pay more attention to the taste and convenience of tea [8]. Previous studies have shown that consumer preferences for tea beverages are influenced by factors such as processing techniques, aroma intensity, and sweetness [9], which mostly focus on single markets; thus, cross-cultural comparisons are lacking. In recent years, the global tea beverage market has experienced rapid growth, leading to increasingly diverse sensory quality demands among consumers. However, existing research has primarily focused on the sensory characteristics of green and black teas, whereas studies of the sensory profiles and preferences for oolong and black oolong teas remain limited [10]. Recent research conducted in South Korea showed that consumers generally assigned lower liking scores to black oolong tea than to oolong tea [11]. This difference in liking is known to cause a contrast effect due to the presentation order and group effects due to the sample composition [12]. The contrast effect has been previously demonstrated in sensory testing [13] and dining [14] and can be minimized by balancing the presentation order in consumer liking tests [12]. However, to the best of our knowledge, the group effect of stimuli has not been demonstrated in previous studies. Group effects may occur when a better-liked sample is tested against several less-liked samples [12]. Consumer familiarity with oolong and black oolong teas differs between China and South Korea, with black oolong tea not being readily available for consumption in Korea. This difference may influence the acceptance of black oolong tea differently. However, comparative studies on the sensory preferences for oolong tea among consumers from different cultural backgrounds are scarce. Cross-cultural comparisons can help identify universal consumer preferences, if existent, and understand how different characteristics impact the preferences of consumers from different tea cultures.

Therefore, this study aimed to identify similarities and differences in preference and sensory perception between two consumer groups differing in their familiarity with samples of oolong and black oolong teas and to evaluate whether assessing these teas in the same session would incur group effects.

## 2. Materials and Methods

### 2.1. Samples

Seven oolong and five black oolong tea samples were obtained via online purchases from retailers in South Korea and China, respectively (Table 1). All but one sample were ready-to-drink (RTD) bottled oolong tea beverages marketed in China, South Korea, and Japan. One sample was concentrated and diluted for consumption. Raw oolong tea leaves were sourced from Fujian Province, China, as indicated on the package labels. All samples were stored in a refrigerator at a temperature of 2 °C. The day before the experiment, the concentrated samples were reconstituted in bottled mineral water (Samdasoo, Jeju Special Self-Governing Province Development Corporation, Jeju, South Korea) in accordance with the manufacturer’s recommended ratio. The mixture was then placed in a 1 L beaker, sealed, and stored in a refrigerator at 2 °C until use. For evaluation purposes, the samples were assigned three-digit random numbers and served in 88.7 mL (3 oz) clear plastic cups (Disposable PET 3 oz Clear Tasting Cup; I-LUX, Icheon-si, Republic of Korea). Samples were labeled with 3-digit random codes for testing under blind conditions to minimize subjective consumer bias based on brand information and product familiarity [15].

### 2.2. Consumer Participants

Consumers aged 19–45 years from Busan, South Korea, were targeted through online school bulletin boards and other digital channels (such as WeChat, Tencent, Shenzhen, China), with specific efforts to exclude individuals with food allergies and those who were pregnant. The recruitment questionnaire utilized SurveyMonkey, an online survey platform, to identify participants. Following a thorough screening process, 102 Chinese (27 males and 75 females) and 100 Korean (25 males and 75 females) consumers were selected to participate in the study, as shown in Table 2. Owing to the limited pool of Chinese consumers residing in Busan, South Korea, age and sex imbalances were inevitable. The age and sex ratios of participants from both cultures were similar. Although sex may affect sensory preferences, previous studies have shown that sex differences generally have little effect on overall preferences in the sensory evaluations of foods and beverages [16]. Both Chinese and Korean consumers resided in Busan, South Korea, and are referred to as Chinese and Korean consumers in this study. A sensory evaluation was conducted using dedicated sensory booths. Before participating, all consumers were informed of this study’s details, and they voluntarily signed a consent form.

### 2.3. Consumer Tests

#### 2.3.1. Questionnaire

The questionnaire employed a 9-point hedonic scale to assess overall sample liking ranging from 1 (dislike extremely) to 9 (like extremely). Further evaluations included liking for appearance (transparency and color), astringency, and aftertaste. Perceived intensity evaluations for oolong tea flavor, bitterness, astringency, and aftertaste were performed using a 9-point intensity scale, with 1 indicating none and 9 indicating extreme.

The intensity rating and the Check-All-That-Apply (CATA) method were used to fully capture consumers’ perceptions of the sensory attributes of the product. The intensity rating is used to quantify the perceived intensity of a few specific attributes, whereas the CATA method is used to identify the key attributes that consumers spontaneously associate with. These two methods can complement each other: the intensity rating provides precise quantitative data, while the CATA method reveals the overall perception pattern of consumers toward a product [17]. The sensory descriptor lexicon for the CATA method was developed based on research presented in other tea sensory-related articles [9,11,18]. Previous studies developed sensory lexicons for other tea types, such as green and blended teas [9,18]. We selected 12 sensory descriptors for oolong tea that had been used in oolong tea-related research and added 8 additional descriptors based on the characteristics of black oolong tea for a total of 20 characteristics for the sensory evaluation experiments. After undergoing translation and verification to ensure accuracy, Chinese and Korean versions of the survey questionnaires were prepared (Table 3). Two terms may be perceived as subjective or ambiguous. Cooling refers to a mild, refreshing, slightly minty or menthol-like feeling in the mouth and throat [19]. Clean describes a mouthfeel without any residual aftertaste or impurities [20]. However, the definitions of these terms were not provided to consumers [11,17]. To ensure consistency in the evaluation process, the order of all descriptive terms was fixed across the questionnaires [17]. The CATA method was employed to identify all the perceivable sensory attributes of the samples in a checkbox format.

Finally, participants were asked about their tea consumption habits, such as tea consumption frequency per week, type of tea consumed, and preferred tea temperature (Table 2).

#### 2.3.2. Test Process

After obtaining approval from the Institutional Review Board, the experiment began in November 2023. To prevent fatigue from evaluating all 11 samples and group effects, the evaluation was divided into 2 sessions. The first session involved seven oolong tea samples (Table 1). The second session included two oolong teas (Suntory Oolong Tea and Ito En Oolong Tea) and five black oolong teas for a total of seven samples. The second session was conducted 1 week after the first session, similar to other consumer tests with a larger number of samples [21,22]. The inclusion of two oolong tea samples (ST and IT) from the first and second sessions was used to confirm the group effect by comparing the results from both sessions. Each evaluation session lasted approximately 1 h. The evaluation was conducted at the Sensory Evaluation Laboratory of Pusan National University. Before the evaluation, each consumer received a brief introduction to the evaluation process and instructions on accessing the corresponding questionnaire. The participants were asked to read and voluntarily sign a consent form before participating in the study. Once consent was obtained, the evaluation commenced. All samples were stored in a refrigerator at 2 °C for 1 day in advance to simulate the conditions of being sold in convenience stores. To minimize subjective bias from brand recognition, all samples were poured into 88.7 mL (3 oz) transparent plastic cups. Each sample was assigned a random three-digit code as an identifier. The same samples used in the second session were assigned different codes to prevent familiarity with the codes from affecting the assessment. The samples were presented in an order determined using William’s Latin Square design [23], with a 5 min interval between each sample. The consumers evaluated seven samples in each session.

Consumers used their own mobile phones for data entry in an online survey format using SurveyMonkey, with Quick Response (QR) codes generated for each sample’s questionnaire. After tasting each sample, consumers scanned the QR code to complete a questionnaire for the corresponding sample. To mitigate any impact on subsequent samples, crackers (Crick Cracker, Crich Nuova Industria Biscotti Crich SPA, Treviso, Italy) and bottled water (Samdasoo 330 mL; Jeju Special Self-Governing Province Development Corporation) were provided for palate cleansing between samples. Consumers were asked to indicate their overall liking of the current sample, select all applicable sensory descriptors (CATA) they could perceive, rate their liking of certain sensory attributes, and assess the intensity of specific characteristics. After completing the evaluation of all samples, consumers scanned another QR code to complete the demographic and consumption questionnaires. Upon completing all questionnaires, consumers were compensated with 10,000 KRW per visit. The QR code was used only as a link to the anonymous questionnaire, and no personally identifiable information (such as name, phone number, or email address) was collected. All data were represented by a consumer number during transmission and storage, ensuring that they could not be traced back to the individual participants. Only aggregated data were used during the data analysis phase to avoid disclosing any information that could identify individuals.

#### 2.3.3. Data Analysis

In both sessions, consumer responses were compared using Fisher’s exact test [24] to determine whether evaluating oolong tea alone or alongside black oolong tea would result in significant differences in selection frequency (α = 0.05). The CATA binary data were initially analyzed using Cochran’s Q test, following Marascuilo’s procedure for comparing pairwise samples and identifying significant differences in sensory attribute selection across tea samples [10]. The Mann–Whitney U test was used to compare sensory perceptions between the consumer groups. Subsequently, a correspondence analysis (CA) was performed to explore the underlying structure of the CATA data and visualize consumer preferences. The RV coefficient test of the CA results was used to evaluate similarities in sensory perception between the two groups [25].

One-way ANOVA was used to compare the acceptability and intensity ratings across samples, and a paired comparison *t*-test was used to assess the overall liking of the same samples in both the first and second sessions.

All statistical analyses were performed using the SAS^®^ Software 9.4 (SAS Institute Inc., Cary, NC, SPSS Inc., Chicago, IL, USA) and the XLstat^®^ Software package (Addinsoft SARL, New York, NY, USA) [10,25].

## 3. Results

### 3.1. Sample Composition Effect Test

In this study, two specific samples, SU and IT, were included in both the first and second evaluations to ensure that all samples from the two evaluation sessions could be analyzed together and to determine the presence of a group effect when evaluating oolong tea alongside black oolong tea. The first evaluation included only oolong tea samples, whereas the second evaluation included five black oolong tea samples. To assess potential bias, a *t*-test was performed on the liking scores of the Chinese and Korean consumers. Cochran’s Q test was used to assess the sensory characteristics of the samples. The results showed no significant differences in the overall liking of the SU and IT samples between the two evaluation sessions for both Chinese and Korean consumers (Table 4). This indicated that the two sessions were comparable and could be analyzed together, and that the inclusion of black oolong tea did not affect the evaluation of oolong tea samples. According to the results of Fisher’s exact test, no significant difference was observed in the selection frequency of sensory characteristics between SU and IT in the options of the two CATA evaluations (Table 5). These findings suggest no group effect on the SU and IT samples tested with black oolong tea, and consumers were consistent in their evaluations across both sessions. Therefore, to simplify the subsequent statistical analyses, the data for SU2 and IT2 were excluded, leaving 12 samples.

### 3.2. Consumer Preferences for Oolong and Black Oolong Teas

The average overall liking scores of both Chinese and Korean consumers were above five on a nine-point scale, indicating the acceptance of both oolong and black oolong teas. The mean liking score ranged between 5.6 and 6.8 for the Chinese consumers and 5.1 and 6.9 for Korean consumers. The most preferred sample among Chinese consumers was black oolong tea (OCB), while Sangaria Oolong tea (SG) and oolong tea (OL) received comparable liking scores. For Korean consumers, SG had the highest liking score, and OCB was the highest-scoring black oolong tea, although its liking score was significantly lower than that of SG (Table 6). It is worth noting that when the 12 samples were counted together, Chinese consumers showed the highest liking for OCB in the black oolong tea category, while Korean consumers showed the highest liking for SG in the oolong tea category. However, when oolong and black oolong teas were analyzed separately, Chinese and Korean consumers preferred SG the most in the oolong tea category and OCB the most in the black oolong tea category. This result shows that although certain differences exist in the overall preference trends in specific categories, the preferences of Chinese and Korean consumers tend to be similar. Regarding the transparent appearance liking, Korean consumers showed a slight dislike for the YCB sample, most likely due to impurities in its concentrated form. In terms of color liking, Chinese consumers did not differ much, as their average ranged between 5.6 and 6.3, whereas Korean consumers differed markedly, with the average color liking ranging from 4.3 to 7.1. Both consumer groups showed similarities in their preferences, with OCB favored for its astringency and aftertaste.

### 3.3. Characteristics Affecting Oolong and Black Oolong Tea Liking

**Figure 1:**
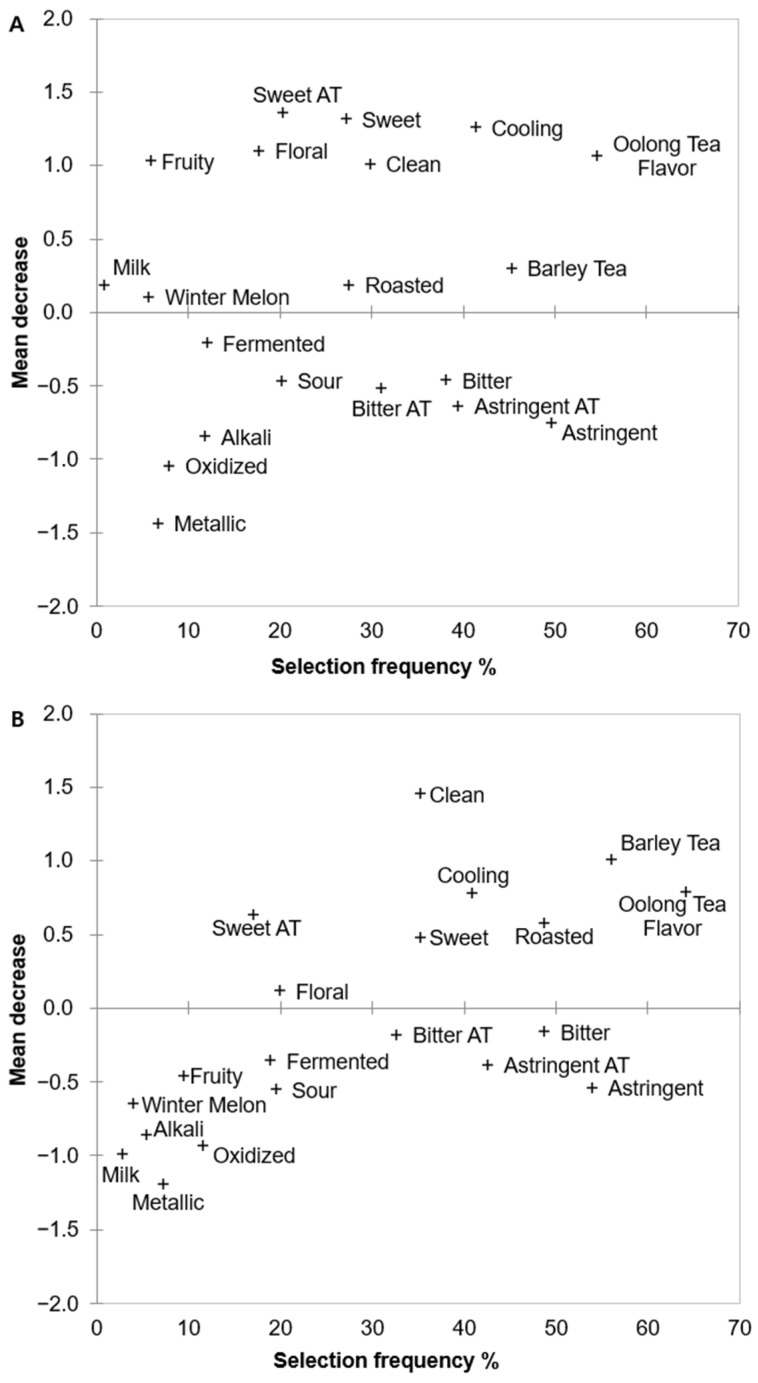
Oolong tea characteristics affecting liking scores of (**A**) Chinese and (**B**) Korean consumers residing in South Korea.

Attributes that positively or negatively impacted overall liking were identified for both Chinese and Korean consumers using the CATA-Penalty analysis (Figure 1), and differences were observed. Chinese consumers positively valued attributes such as a sweet aftertaste, sweetness, cooling, oolong tea flavor, cleanliness, and barley tea flavor. Negative effects included astringency, astringent aftertaste, bitterness, bitter aftertaste, and sourness. Sweet aftertaste had the most significant positive impact, reflecting the Chinese cultural concept of “Hui Gan” [26]. Although slightly different from the sweet aftertaste, it refers to the brief astringency perceived upon entry into the mouth. Over time, the astringent sensation fades, and a slight sweetness spreads from the throat after saliva is secreted. For Korean consumers, positive attributes included cleanliness, barley and oolong tea flavors, cooling, roasted flavor, and sweetness, with astringency and astringent aftertaste being negative attributes. Cleanliness was the most significant positive attribute and aligned with the Korean preference for heavily roasted teas [27]. Overall, both consumer groups valued sweetness, cleanliness, and barley tea flavor but disliked astringency and astringent aftertastes. This result was similar to that reported by Jaeger et al. [28]. Bitterness is an important negative factor affecting overall consumer liking. However, according to a 2010 study of green tea, Korean consumers did not like green tea without bitterness, but “too bitter” and “not bitter enough” were other reasons that led to a decrease in liking [29]. As CATA only captures the consumer noting the characteristics, differentiating between bitterness, as an important sensory characteristic, and being too bitter is difficult. These attributes are crucial for determining liking for oolong and black oolong teas. Korean consumers indicated that samples without bitterness were not green teas [30].

### 3.4. Sensory Characteristics of Oolong and Black Oolong Teas

#### 3.4.1. Intensity Rating

Oolong tea flavor, bitterness, astringency, and aftertaste were rated using a nine-point intensity scale, and their mean scores are shown in Table 7. Although the score range differed slightly in terms of the intensity of oolong tea flavor, both Chinese and Korean consumers agreed that ST had the highest intensity among oolong teas, while FNB had the highest intensity among black oolong teas. KN and YCB were rated lowest in intensity for oolong and black oolong teas, respectively. This could be attributed to the dilution of YCB, which might have lowered its intensity compared with that of the other RTD samples. The perception of flavor intensity was similar between the two groups, although cultural differences were observed in understanding the intensity.

#### 3.4.2. Correspondence Analysis (CA)

**Figure 2:**
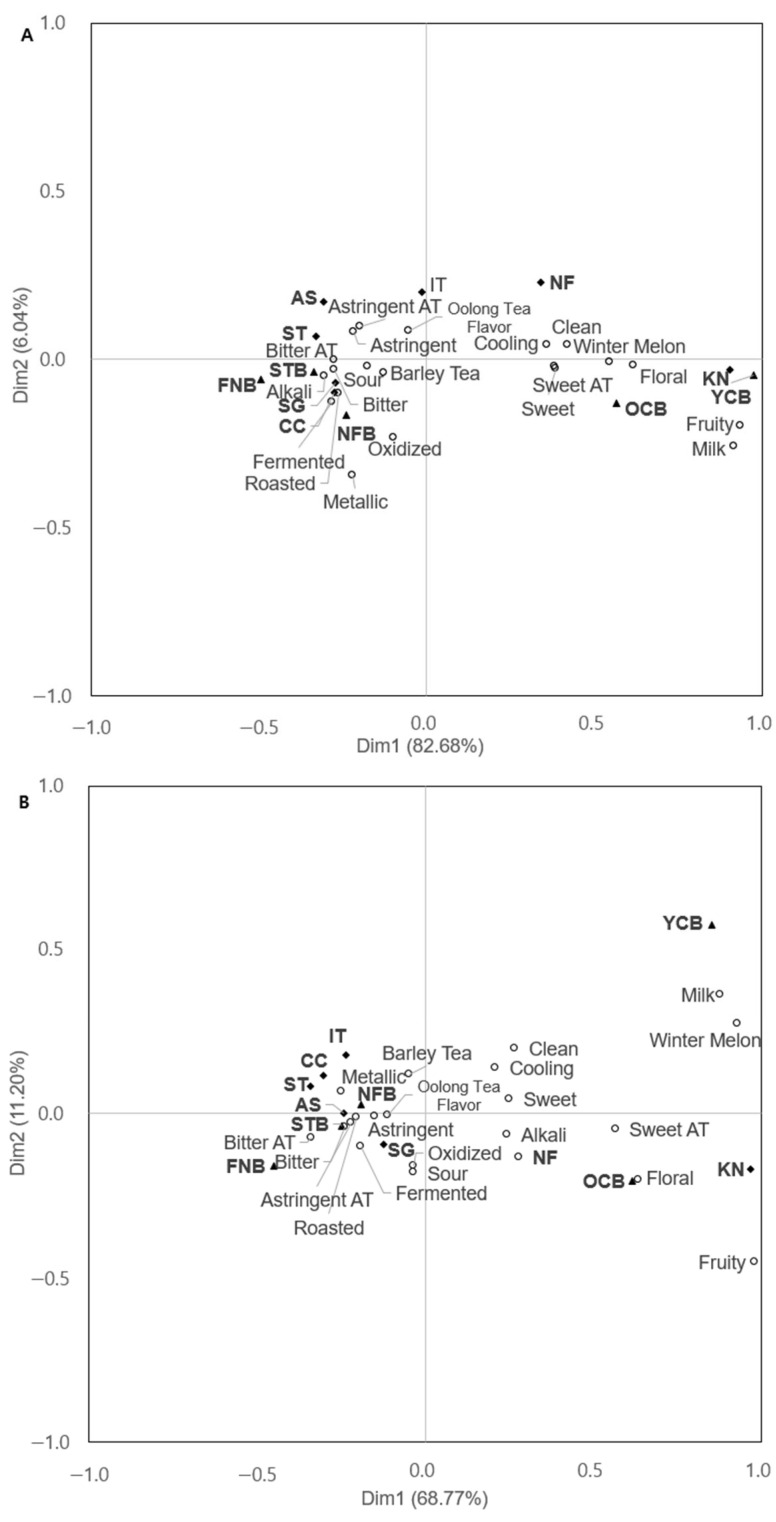
Correspondence analysis biplots of 12 oolong and black oolong tea samples evaluated by (**A**) Chinese and (**B**) Korean consumers residing in South Korea. ST, Suntory Oolong tea; SG, Sangaria Oolong tea; CC, Coca-Cola Oolong tea; NF, Oriental Leaf Oolong tea; AS, Asahi Oolong tea; KN, Golden Flowers Oolong tea; IT, Ito En Oolong tea; OCB, OCOCO; YCB, YU-CHA; FNB, Tominaga; NFB, Oriental Leaf Black Oolong; STB, Suntory Black Oolong tea.

CA was performed on the CATA frequency data, revealing significant divergence in sensory attributes among the NF, KN, OCB, and YCB samples (Figure 2). For Chinese consumers, oolong tea samples were associated with attributes such as “winter melon”, “oolong tea”, “sweet aftertaste”, and “clean”. Black oolong teas were characterized with “oxidized”, “metallic”, and “fermented” attributes. Similarly, for Korean consumers, black oolong tea samples were associated with “alkali”, “fermented”, and “oxidized” attributes, while oolong tea samples were linked to “sweet”, “barley tea”, and “clean” attributes. The oolong tea flavor was not a differentiating attribute, probably because all samples had an oolong tea flavor (Table 7). This may be because Korean consumers have fewer opportunities to be exposed to oolong tea and may find it difficult to understand its flavor. For Chinese consumers, sensory attributes such as “bitter”, “astringent”, and “roasted” contributed little to distinguishing the two teas, as these attributes were present in most samples to varying degrees, and Chinese consumers may consider these attributes as key attributes in oolong tea (Figure 2). In the RV coefficient analysis of data from the Chinese and South Korean consumer groups, an RV value of zero indicates no correlation between the two matrixes, whereas a value of one represents complete consistency [31]. The RV coefficient for the samples was 0.952 (*p* < 0.0001), indicating high similarity in sample comparisons between Chinese and Korean consumers. The RV coefficient of the attributes was 0.862, which was also significant (*p* < 0.0001), suggesting that the sensory perception was similar between the two groups. Overall, these results demonstrate a high level of similarity in the sensory evaluations between Chinese and Korean consumers.

#### 3.4.3. Perception Differences Determined Using CATA and Intensity Data

The mean intensity ratings and corresponding CATA frequencies for four attributes—oolong tea flavor, bitter taste, astringency, and aftertaste—were reported by Chinese and Korean consumers. CATA selections and corresponding intensity ratings were compared based on whether participants checked each term (Table 8). Although the mean rating and CATA frequency summation for each sample were similar, the differences in mean ratings between Chinese and Korean consumers based on CATA selections did not differ. Among Chinese consumers, 11 samples showed a significantly higher intensity of oolong tea flavor when the term was selected. In contrast, among Korean consumers, only seven samples showed a significantly higher intensity when the term was selected, and the mean differences were much smaller compared with those for Chinese consumers’ evaluations. For example, the mean IT rating differed significantly by 1.4 for Chinese consumers, but for Korean consumers, the rating difference was only 0.3 and not significant. The difference was more obvious in the bitterness and astringency results. Every sample was rated higher in bitterness by Chinese consumers when bitterness was selected in the CATA question. However, for Korean consumers, five samples showed no significant differences in intensity ratings, among which four were black oolong tea samples. For the astringency evaluation, seven of the twelve samples did not show a significant difference in intensity rating between checking and not checking the CATA question for Koreans. Four of these were black oolong teas.

**Figure 3:**
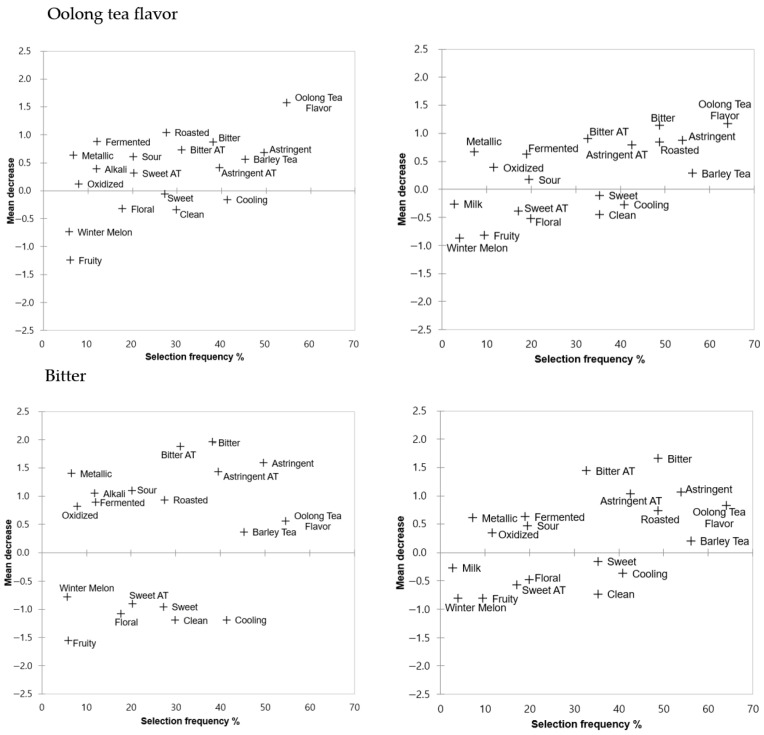

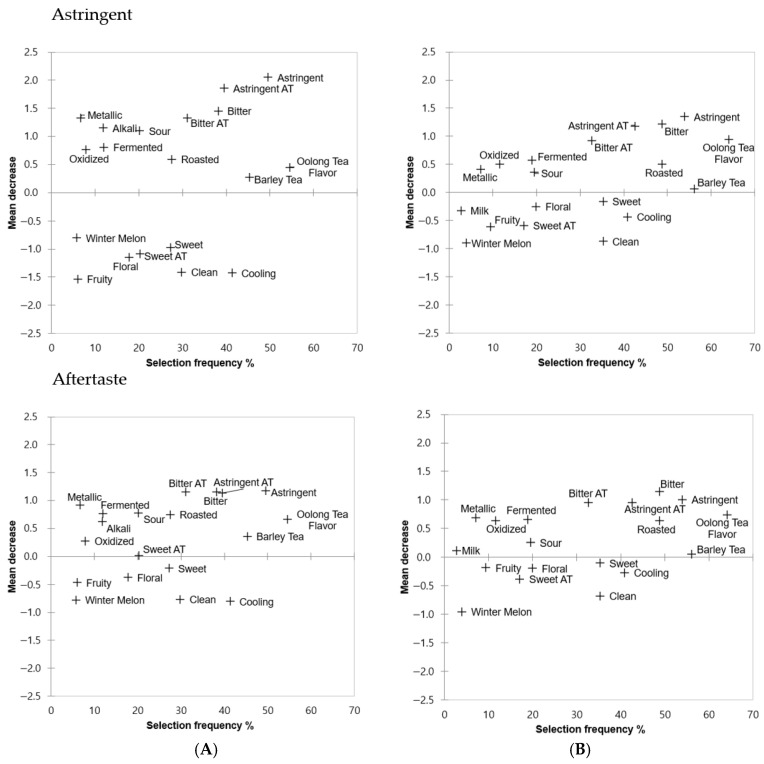
Oolong tea attributes affecting the intensity ratings of (**A**) Chinese and (**B**) Korean consumers residing in South Korea. The *y*-axis in all figures represents the average effect of liking-based selections on the intensity score of each attribute. The *x*-axis shows the proportion of participants who selected these attributes.

Penalty analyses were conducted using the intensity ratings and CATA selections for the same corresponding attributes (Figure 3). This “characteristic CATA-Penalty” analysis shows the aggregated influence of selected CATA attributes on a single intensity rating, similar to the CATA-Penalty approach for overall liking. For oolong tea flavor intensity, Chinese consumers who selected “oolong tea flavor” rated it 1.6 points higher than those who did not. Other attributes associated with a mean decrease in intensity included “roasted” (−1.0), “bitter” (−0.9), “astringent” (−0.7), “sour” (−0.6), and “barley tea flavor” (−0.6). Korean consumers who selected “oolong tea flavor” had a rating that was 1.2 points higher than those who did not. The mean decrease in intensity for “bitter” was 1.1, followed by “astringent” at 0.9, “bitter aftertaste” at 0.9, “astringent aftertaste” at 0.8, “roasted” at 0.8, and “barley tea flavor” at 0.3.

Similarly, attributes with mean decreases in bitterness and astringency differed in degree and/or importance for Chinese and Korean consumers. Notably, for aftertaste, attributes associated with a mean decrease were similar, with astringent, bitter, bitter aftertaste, astringent aftertaste, roasted, and oolong tea flavor contributing positively. Sweet aftertaste did not show a significant mean decrease.

### 3.5. Consumption Habits

**Figure 4:**
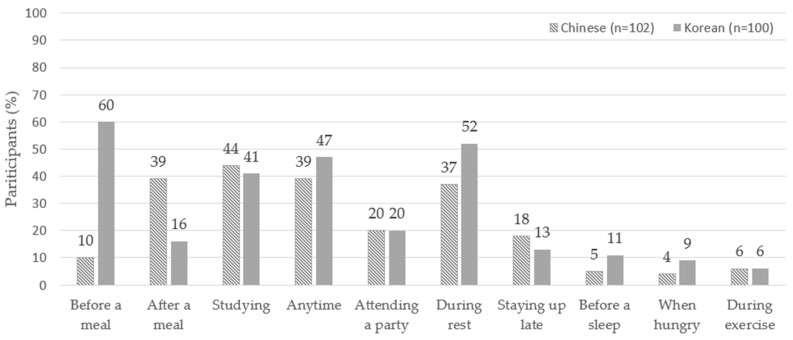
Timing of tea beverage consumption.

Demographic questions included tea consumption habits. The results revealed that Chinese consumers had a higher frequency of oolong tea consumption than did Koreans, likely due to the origin of oolong tea in China. Approximately 30% of consumers from both groups preferred cold tea. Notably, 11.8% of Chinese consumers preferred room-temperature tea, whereas only 3% of Korean consumers were likely to be influenced by the prevalent sale of chilled bottled tea in Korea. The survey also highlighted cultural differences in consumption timing (Figure 4). Korean consumers often drank tea before meals, whereas Chinese consumers preferred tea after meals. This difference may stem from the habitual consumption of barley tea in Korea and the tradition of tea as a digestive aid in China [30,32]. Conversely, the Chinese tradition of consuming tea post-meal [33] underscores the cultural inclination toward tea as a digestive aid. Cultural values reflect what society considers important and desirable, thus forming unwritten rules and expectations regarding social behavior. These underlying rules influence the purchasing decisions and consumption patterns of consumers [33]. These findings emphasize the significant influence of cultural background on tea preferences and consumption habits.

## 4. Discussion

This study explored the cross-cultural differences in the preferences for and sensory perceptions of bottled oolong and black oolong teas between Chinese and Korean consumers. Currently, RTD black oolong tea beverages are available only in Japan and China. RTD oolong tea is also difficult to find in readily accessible stores in Korea, resulting in Korean consumers having limited exposure to oolong teas other than those brewed from tea leaves. Although the study was conducted in Korea, almost twice as many Chinese consumers drank oolong tea compared with Korean consumers, despite similar green and black tea consumption rates. Based on overall liking, liking of appearance (transparency and color), astringency, and aftertaste did not differ significantly between Chinese and Korean participants. Based on the CA biplot derived from CATA data, a high RV coefficient indicated similarity in evaluations. The intensity ratings of oolong tea flavor, bitterness, astringency, and aftertaste also did not show much difference between Chinese and Korean consumer perceptions. The intensity scores from Chinese consumer groups were generally only 0.1–0.2 points higher than those of Korean consumers. However, when the intensity ratings of these four attributes were evaluated alongside CATA responses, a difference emerged between Chinese and Korean consumers. Chinese consumers reported higher intensity ratings when selecting the corresponding CATA terms. However, Korean consumers showed little variation in the intensity rating of black oolong tea, regardless of whether they selected the corresponding attributes in the CATA question. A lack of familiarity may have influenced this result, highlighting the variations in sensory perception influenced by cultural background [34,35]. When Chinese consumers choose among different global product brands, brand awareness is the most influential factor driving their purchase intention, followed by cultural values and social influence. Maintaining product consistency with local cultural norms can increase consumer acceptance. This is confirmed by the consumption habits of Chinese and Korean consumers [36,37].

Studies have shown that consumers unfamiliar with tea beverages may describe disliked samples simply as “bitter” or conflate astringency with bitterness, using “bitterness” to represent the combined sensation of both traits [38]. Future research should address the negative impact of bitterness on overall liking evaluations. Most Chinese consumers prefer tea beverages with sweet, mild, and fruity profiles, favoring products with low bitterness and astringency [39]. These findings align with the results of the present study, where sweet taste and sweet aftertaste positively influenced overall liking in the selected Chinese consumer group, whereas astringency served as a primary negative factor. Korean consumers prefer green tea, which has a stronger “sweet taste” and roasting-related flavors such as “roasted barley” and “burned leaf” [28]. This observation corroborates the unique positive association between roasted flavors and overall liking among the surveyed Korean consumers. However, unlike Chinese consumers, Korean consumers do not perceive bitterness as detrimental to their overall liking of oolong and black oolong teas, which may be attributed to their habitual consumption of green teas [40] or coffee [41,42] with pronounced bitterness.

Asking consumers to provide both CATA responses and intensity ratings for all characteristics may be burdensome for them and may require several evaluation sessions [43]. If the important characteristics can be predetermined by researchers or qualitative studies [17], a short list of characteristics can be rated for intensity in the same evaluation session. Additionally, *t*-test evaluations of intensity ratings between individual attributes that were checked and not checked for each sample may be cumbersome [43]. Utilizing the CATA-Penalty approach for intensity measurements may provide similar insights into the data, but it would be easier for researchers to conduct the analysis. However, the just-about-right approach may be more suitable when attributes exhibit optimal intensity. The CATA-Penalty may not be able to demonstrate the contribution of these attributes to overall liking.

The results of the tea consumption occasions (Figure 4) showed cultural differences in tea consumption timing between Chinese and South Korean consumers. Chinese consumers tend to consume tea after meals, whereas South Korean consumers tend to do so before meals. These differences may be attributed to the combined effects of historical, social, and economic factors. In China, tea drinking has been a social activity since ancient times [44], and doing so after meals provides an opportunity for communication between family and friends [1]. In Korea, tea consumption before meals are associated with personal health and relaxation [45]. The diversity and marketing strategies of China’s tea industry may have strengthened the habit of drinking tea after meals, while the unique positioning of the Korean tea market, which prefers cold tea, may have promoted the popularity of tea consumption before meals. These results may provide new perspectives for future research, such as exploring the diversity of tea consumption behavior and its driving factors in other cultural contexts.

This study has a few limitations. One limitation is the sex imbalance, with a higher proportion of female participants among both Chinese and South Korean consumers. Although sex may affect sensory preferences, previous studies have shown that sex differences generally have little effect on overall preferences in the sensory evaluation of foods and beverages [16,46], and when differences exist, they are confounded by consumption frequency. Therefore, we believe that this sex imbalance did not significantly affect the generalizability of our results. Future research should aim to achieve a more balanced sex distribution to enhance the generalizability of the findings.

Although Chinese consumers, similar to their Korean counterparts, were selected from the young demographic of university students, they may not be true representatives of Chinese consumers. This group included only Chinese students studying in South Korea. The duration of their stay in South Korea varied, including those who had just arrived and those who had lived there for over 5 years. These varying degrees of exposure to the Korean diet and lifestyle habits may have influenced the evaluation results. Some individuals may have acculturated and changed their consumption habits. Koreans drink most beverages iced [47], whereas Chinese consumers tend to choose the traditional hot water brew of tea [48]. However, both the Chinese and Korean participants in the current study showed similar preferred tea consumption temperatures. In addition, owing to China’s large geographical area and population, dietary habits and consumption preferences vary significantly among consumers from different regions. To reduce this bias, future studies should directly recruit participants from different regions of China to reflect the diversity of Chinese consumers more fully. Despite this limitation, we emphasize that the use of convenient sampling in cross-cultural consumer tests remains valuable for obtaining initial feedback, enhancing the understanding of cultural similarities and differences [49], and supporting the effective planning of comprehensive cross-cultural studies. Cross-regional comparative studies could further reveal the impacts of acculturation and regional differences on tea consumption preferences.

## 5. Conclusions

This study demonstrated the preferences for oolong and dark oolong teas among young Chinese and Korean university students residing in Busan, South Korea. It highlights the profound influence of cultural background on tea consumption habits and sensory perceptions. Both groups of young consumers preferred cold tea beverages; however, Chinese consumers favor post-meal tea consumption, whereas South Korean consumers lean toward pre-meal consumption. Sensory evaluations showed that Chinese consumers prioritized sweeter and less bitter teas, aligning with their emphasis on the social attributes of tea. In contrast, South Korean consumers exhibited a higher tolerance for bitterness and a distinct preference for roasted flavor profiles. For manufacturers and marketers, these findings underscore the need to tailor product formulations and marketing strategies to suit cultural norms. Developing region-specific flavor profiles (e.g., low-astringency options for China and roasted flavor variants for South Korea), diversifying temperature-based offerings (cold and room-temperature variants), and aligning promotional messaging with consumption contexts (post-meal vs. pre-meal positioning, perhaps differing in the quantity of the beverage) could enhance market acceptance.

Future research should address China’s regional diversity by recruiting participants directly from different areas and minimizing cultural adaptation biases to improve product localization efforts. By integrating cultural insights into product design and branding, stakeholders can better meet consumer expectations and foster cross-cultural appeal while respecting traditional customs. Another recommendation for future investigations is to define the CATA terms for consumer use. Although the CATA method has been widely used, no research has been conducted to determine whether consumers understand the sensory terms presented, presenting a scope for future studies.

## Figures and Tables

**Table 1:** Sample information.

Abb.	English Name	Korean Name	Chinese Name	Brand	Origin
ST	Suntory Oolong tea	산토리 우롱차	三得利乌龙茶	Suntory	Japan
SG	Sangaria Oolong tea	산가리아 우롱차	三佳利乌龙茶	Iyemon Tea House	Japan
CC	Coca-Cola Oolong tea	코카콜라 우롱차	可口可乐乌龙茶	Coca-Cola	Japan
NF	Oriental Leaf Oolong tea	농푸 동방수예 우롱차	东方树叶乌龙茶	Nongfu Spring	China
AS	Asahi Oolong tea	아사히 우롱차	朝日乌龙茶	ASAHI	Japan
KN	Golden Flowers Oolong tea	금빛 우롱차	金花乌龙茶	Keumnong Foods	Korea
IT	Ito En Oolong tea	이토엔 우롱차	伊藤 园乌龙茶	Ito En	Japan
OCB	OCOCO	오코코 흑우롱차	OCOCO黑乌龙茶	OCOCO	China
YCB ‡	YU-CHA	농축 흑우롱차	浓缩黑乌龙	You Cha	Japan
FNB	Tominaga	토미나가	富永黑乌龙茶	Funei	Japan
NFB	Oriental Leaf Black Oolong	농푸 동방수예 흑우롱차	东方树叶黑乌龙茶	Nongfu Spring	China
STB	Suntory Black Oolong	산토리 흑우롱차	三得利黑乌龙茶	Suntory	Japan

Abbreviations were obtained from the first two syllables of the product brand. Samples ending in B indicate black oolong tea. ‡ YCB is a concentrated product; it was diluted 20 times of the volume with drinking water (330 mL, Samdasoo Jeju Special Self-Governing Province Development Corporation, Jeju, Republic of Korea) according to the manufacturer’s recommended method.

**Table 2:** Consumers’ demographic information and tea consumption habits.

	Chinese (*n* = 102) *n* (%)	Korean (*n* = 100) *n* (%)
Sex		
Male	27 (26.4)	25 (25.0)
Female	75 (73.5)	75 (75.0)
Age (years)		
19–25	63 (61.8)	78 (78.0)
26–30	34 (33.3)	12 (12.0)
31–35	4 (3.9)	4 (4.0)
36–40	1 (1.0)	2 (2.0)
41–45	0 (0.0)	4 (4.0)
Tea consumption frequency per week		
0	10 (9.8)	21 (21.0)
1	37 (36.2)	39 (39.0)
2	18 (17.6)	22 (22.0)
3	13 (12.7)	9 (9.0)
>4	24 (23.5)	9 (9.0)
Type of tea consumed		
Black Tea	65 (63.7)	73 (73.0)
Green Tea	55 (53.9)	53 (53.0)
Oolong Tea	53 (52.0)	26 (26.0)
Other *	32 (31.4)	45 (45.0)
Preferred drinking temperature of tea		
Cold	32 (31.4)	30 (30.0)
Hot	45 (44.1)	43 (43.0)
Room temperature	12 (11.8)	3 (3.0)
No Preference	13 (12.7)	24 (24.0)

* Other categories include non-traditional tea types such as herbal teas, flower teas, fruit teas, etc. These teas are classified as “other” due to their unique processing methods and flavor profiles to distinguish them from the main tea types, such as green, black, and oolong teas.

**Table 3:** Sensory terminology provided to consumers ^†^.

English	Chinese	Korean
Bitter	苦味	쓴맛
Sour	酸味	신맛
Sweet	甜味	단맛
Astringent	涩味	떫은맛
Cooling	清爽的	시원한
Metallic	金属味	금속 맛
Alkali	碱味	알칼리 맛
Barley Tea	大麦茶味	보리차 맛
Fermented	发酵味	발효 맛
Floral	花香味	꽃향
Fruity	水果味	과일 맛
Rosted	烘烤味	구운 맛
Milk	牛奶味	우유 맛
Oolong Tea Flavor	乌龙茶味	우롱차 맛
Oxidized	氧化味	산화 맛
Winter Melon	冬瓜味	동과 맛
Bitter Aftertaste	回味苦	쓴 후미
Sweet Aftertaste	回味甜	단 후미
Astringent Aftertaste	回味涩	떫은 후미
Clean	干净的	깔끔한

^†^ For Chinese consumers, all three translations were presented. For Korean consumers, Korean and English translations were presented.

**Table 4:** Paired *t*-test comparing the overall liking evaluations of the same samples in different evaluation sessions.

	ST	ST2	*p*-Value	IT	IT2	*p*-Value
Chinese consumers	6.02	6.12	0.614	6.46	6.46	0.9099
Korean consumers	6.23	6.35	0.599	6.65	6.93	0.153

ST, Suntory Oolong tea; IT, Ito En Oolong tea.

**Table 5:** Fisher’s exact test of the attribute frequency of the same samples in different evaluation sessions.

	ST		ST2		IT		IT2	
	*n*	%	*n*	%	*n*	%	*n*	%
Korean consumers residing in South Korea (*n* = 102)								
Sweet	38	37.3	30	29.4	39	38.2	35	34.3
Bitter	60	58.8	56	54.9	60	58.8	49	48.0
Sour	15	14.7	16	15.7	20	19.6	16	15.7
Astringent	64	62.7	69	67.6	63	61.8	65	63.7
Barley tea	62	60.8	64	62.7	64	62.7	67	65.7
Roasted	60	58.8	49	48.0	59	57.8	53	52.0
Oxidized	9	8.8	6	5.9	12	11.8	12	11.8
Floral	12	11.8	8	7.8	7	6.9	12	11.8
Fruity	0	0.0	3	2.9	4	3.9	6	5.9
Milk	2	2.0	0	0.0	2	2.0	2	2.0
Fermented	20	19.6	12	11.8	21	20.6	19	18.6
Oolong tea flavor	76	74.5	83	81.4	76	74.5	74	72.5
Metallic	8	7.8	2	2.0	6	5.9	4	3.9
Winter melon	3	2.9	2	2.0	2	2.0	2	2.0
Sweet aftertaste	8	7.8	13	12.7	22	21.6	17	16.7
Bitter aftertaste	45	44.1	40	39.2	39	38.2	31	30.4
Astringent aftertaste	54	52.9	53	52.0	52	51.0	50	49.0
Alkali	2	2.0	2	2.0	2	2.0	4	3.9
Cool	39	38.2	46	45.1	32	31.4	37	36.3
Clean	26	25.5	46	45.1	33	32.4	42	41.2
*p*-value	0.2748				0.9905			
Chinese consumers residing in South Korea (*n* = 100)								
Sweet	19	19.0	21	21.0	19	19.0	37	37.0
Bitter	54	54.0	55	55.0	37	37.0	45	45.0
Sour	21	21.0	22	22.0	13	13.0	20	20.0
Astringent	65	65.0	74	74.0	56	56.0	58	58.0
Barley tea	45	45.0	57	57.0	46	46.0	56	56.0
Roasted	32	32.0	36	36.0	28	28.0	28	28.0
Oxidized	8	8.0	6	6.0	5	5.0	9	9.0
Floral	5	5.0	12	12.0	16	16.0	13	13.0
Fruity	0	0.0	3	3.0	4	4.0	5	5.0
Milk	0	0.0	1	1.0	0	0.0	1	1.0
Fermented	14	14.0	22	22.0	7	7.0	9	9.0
Oolong tea flavor	71	71.0	65	65.0	68	68.0	63	63.0
Metallic	9	9.0	10	10.0	2	2.0	6	6.0
Winter melon	3	3.0	4	4.0	4	4.0	7	7.0
Sweet aftertaste	18	18.0	25	25.0	21	21.0	31	31.0
Bitter aftertaste	40	40.0	40	40.0	34	34.0	33	33.0
Astringent aftertaste	51	51.0	46	46.0	47	47.0	38	38.0
Alkali	14	14.0	17	17.0	7	7.0	7	7.0
Cool	27	27.0	29	29.0	47	47.0	48	48.0
Clean	21	21.0	18	18.0	36	36.0	34	34.0
*p*-value	0.9205				0.7165			

Twenty attributes were used for the CATA evaluation. Statistical significance was set at *p* < 0.05. ST, Suntory Oolong tea; IT, Ito En Oolong tea.

**Table 6:** Oolong tea liking comparisons of Chinese and Korean consumers residing in South Korea.

	Overall Liking		Transparent Appearance Liking		Color Liking		Astringency Liking		Aftertaste Liking	
Chinese consumers residing in South Korea										
AS	5.9	c	6.4	bc	6.1	ab	5.5	fg	5.5	cd
CC	5.7	c	6.0	efg	6.1	ab	5.7	cdefg	5.6	cd
IT	5.8	c *	6.1	def	6.2	ab	5.6	defg	5.6	cd
KN	5.9	c	6.6	ab	6.2	ab	6.0	abcd	6.0	bc
NF	6.4	ab	6.8	a	6.3	ab	6.2	abc	6.1	bcd
SG	6.5	a	6.3	bcde	6.3	ab	6.0	abc	6.2	bcd
ST	6.0	c	6.0	def	6.1	ab	5.6	efg	5.7	bcd
OCB	6.8	a *	6.3	bcde	6.4	a	6.4	a	6.6	a *
NFB	6.0	bc *	6.4	bcd	6.3	ab	5.7	cdef	5.8	bcd *
STB	5.8	c	5.6	g	5.6	c	5.4	fg	5.4	d
FNB	5.6	c	5.8	fg	5.9	bc	5.3	g	5.4	d
YCB	5.7	c *	6.1	cdef *	6.1	ab *	5.9	bcde	5.7	bcd
LSD	0.43		0.40		0.43		0.40		0.44	
*p*-value	<0.0001		<0.0001		0.0315		<0.0001		<0.0001	
Korean consumers residing in South Korea										
AS	6.3	b	6.7	ab	6.5	bc	5.5	cd	5.7	bcd
CC	5.9	bc	6.6	ab	6.2	cd	5.9	bc	5.9	ab
IT	6.0	b *	6.8	a	6.7	ab	5.7	cd	5.7	bc
KN	5.5	cd	5.8	c	4.9	f	5.9	bc	5.7	bc
NF	6.1	b	6.6	ab	6.0	d	5.9	bc	5.9	ab
SG	6.9	a	6.9	a	7.1	a	6.1	ab	6.3	a
ST	6.2	b	6.4	b	6.2	cd	5.9	bc	5.9	ab
OCB	6.2	b *	6.4	b	6.1	cd	6.4	a	6.2	a *
NFB	5.5	cd *	6.6	ab	6.5	bc	5.7	bcd	5.3	cd *
STB	5.8	bc	5.9	c	5.4	e	5.7	bcd	5.7	bc
FNB	5.4	cd	5.8	c	5.3	ef	5.3	d	5.2	d
YCB	5.1	d *	4.5	d *	4.3	g *	5.8	bcd	5.5	bcd
LSD	0.47		0.38		0.43		0.43		0.49	
*p*-value	<0.0001		<0.0001		<0.0001		0.0005		<0.0001	

An independent *t*-test was used to determine significant differences between the evaluations of the Chinese and Korean consumers residing in South Korea. A *p*-value less than 0.05, marked with an asterisk (*), indicated a significant difference. LSD is the least significant difference applied for pairwise comparisons where ANOVA is significant. ^a–g^ Mean values that do not share the same alphabet represent a significant difference (*p* < 0.05). ST, Suntory Oolong tea; SG, Sangaria Oolong tea; CC, Coca-Cola Oolong tea; NF, Oriental Leaf Oolong tea; AS, Asahi Oolong tea; KN, Golden Flowers Oolong tea; IT, Ito En Oolong tea; OCB, OCOCO; YCB, YU-CHA; FNB, Tominaga; NFB, Oriental Leaf Black Oolong; STB, Suntory Black Oolong tea.

**Table 7:** Oolong tea sensory property intensity perception comparisons of Chinese and Korean consumers residing in South Korea.

	Oolong Tea Flavor Intensity		Bitterness Intensity		Astringency Intensity		Aftertaste Intensity	
Chinese consumers residing in South Korea								
AS	5.7 ± 1.52	cd	5.5 ±1.65	bcd	6.0 ± 1.62	ab	6.2 ± 1.34	bc
CC	5.7 ± 1.79	cd	5.2 ± 1.98	cd	5.1 ± 1.84	de	5.6 ± 1.66	dc
IT	6.1 ± 1.58	abc	5.3 ± 1.80	cd	5.6 ± 1.85	c	6.0 ± 1.80	bc
KN	3.6 ± 1.58	e	2.7 ± 1.80	f	3.1 ± 1.85	f	3.9 ± 1.80	e
NF	5.4 ± 1.70	d	4.2 ± 1.66	e	4.7 ± 1.85	e	5.4 ± 1.63	dc
SG	6.0 ± 1.75	abc	5.0 ± 1.66	d	5.3 ± 1.57	cd	5.6 ± 1.59	cd
ST	6.4 ± 1.71	abc	5.8 ± 1.70	b	6.2 ± 1.51	a	6.3 ± 1.56	ab
OCB	6.1 ± 1.57	abc	4.4 ± 1.67	e	4.8 ± 1.74	e	5.9 ± 1.46	bc
NFB	5.9 ± 1.64	bc	5.5 ± 1.62	bcd	5.7 ± 1.66	bc	6.2 ± 1.55	ab
STB	6.1 ± 1.62	abc	5.8 ± 1.61	b	6.2 ± 1.62	a	6.2 ± 1.57	ab
FNB	6.2 ± 1.65	abc	6.2 ± 1.70	a	6.4 ± 1.60	a	6.6 ± 1.58	a
YCB	3.4 ± 1.78	e	2.6 ± 1.49	f	2.7 ± 1.57	f	3.5 ± 1.72	f
LSD	0.41		0.42		0.43		0.40	
*p*-value	<0.0001		<0.0001		<0.0001		<0.0001	
Korean consumers residing in South Korea								
AS	6.0 ± 1.49	abc	5.4 ± 1.82	b	5.7 ± 1.96	ab	5.9 ± 1.67	bc
CC	5.8 ± 1.80	bc	5.0 ± 1.90	cd	5.0 ± 2.02	cd	5.8 ± 1.87	bc
IT	6.0 ± 1.58	abc	5.0 ± 1.90	bc	5.3 ± 1.93	bcd	5.8 ± 1.50	bc
KN	3.4 ± 1.46	e	3.0 ± 1.60	e	3.4 ± 2.00	f	4.2 ± 1.80	e
NF	5.1 ± 1.66	d	4.3 ± 1.88	d	4.9 ± 1.87	d	5.1 ± 1.65	d
SG	5.9 ± 1.71	bc	5.2 ± 1.83	bc	5.4 ± 1.84	bc	5.6 ± 1.67	c
ST	6.3 ± 1.60	a	5.4 ± 1.78	b	5.7 ± 1.87	ab	5.8 ± 1.68	bc
OCB	5.6 ± 1.64	c	4.2 ± 1.70	d	4.4 ± 1.87	e	5.6 ± 1.54	c
NFB	5.8 ± 1.52	bc	5.1 ± 1.87	bc	5.2 ± 1.86	cd	5.7 ± 1.69	bc
STB	6.1 ± 1.72	ab	5.2 ± 1.94	bc	5.4 ± 2.03	bc	6.0 ± 1.76	b
FNB	6.3 ± 1.54	a	6.0 ± 1.80	a	6.1 ± 1.73	a	6.5 ± 1.43	a
YCB	2.9 ± 1.53	f	2.5 ± 1.26	f	2.6 ± 1.35	g	3.2 ± 1.53	f
LSD	0.38		0.42		0.46		0.40	
*p*-value	<0.0001		<0.0001		<0.0001		<0.0001	

LSD is the least significant difference applied for pairwise comparisons where ANOVA is significant. ^a–g^ Mean values that do not share the same alphabet represent a significant difference (*p* < 0.05). ST, Suntory Oolong tea; SG, Sangaria Oolong tea; CC, Coca-Cola Oolong tea; NF, Oriental Leaf Oolong tea; AS, Asahi Oolong tea; KN, Golden Flowers Oolong tea; IT, Ito En Oolong tea; OCB, OCOCO; YCB, YU-CHA; FNB, Tominaga; NFB, Oriental Leaf Black Oolong; STB, Suntory Black Oolong tea.

**Table 8:** Oolong tea flavor intensity perception comparisons of Chinese and Korean consumers residing in South Korea.

	Chinese Consumers (*n* = 102)						Korean Consumers (*n* = 100)					
	Mean Rating	CATA Freq.	Mean Rating (*n*)		*t*-Value	*p*-Value	Mean Rating	CATA Freq.	Mean Rating (*n*)		*t*-Value	*p*-Value
Sample			Not Checked	Checked					Not Checked	Checked		
Oolong Tea Flavor												
ST	6.4	71	5.4 (31)	6.8 (71)	3.597	0.0008	6.3	78	5.4 (22)	6.5 (78)	2.891	0.007
IT	6.0	68	5.1 (34)	6.5 (68)	3.909	0.0003	5.9	82	5.7 (18)	6.0 (82)	0.593	0.5581
CC	5.8	50	5.0 (52)	6.5 (50)	4.757	<0.0001	5.8	64	5.2 (36)	6.1 (64)	2.118	0.0385
NF	5.4	67	4.2 (35)	6.1 (67)	6.069	<0.0001	5.1	67	4.6 (33)	5.3 (67)	2.048	0.0453
AS	5.7	55	5.1 (47)	6.3 (55)	4.074	<0.0001	6.0	75	5.3 (25)	6.2 (75)	2.814	0.0074
KN	3.6	33	3.2 (69)	4.4 (33)	3.176	0.0023	3.4	40	3.3 (60)	3.6 (40)	0.961	0.3393
SG	6.1	59	5.5 (43)	6.5 (59)	3.132	0.0024	6.0	69	5.4 (31)	6.2 (69)	2.581	0.0127
OCB	6.1	58	5.4 (44)	6.5 (58)	3.688	0.0004	5.6	57	5.0 (43)	6.1 (57)	3.290	0.0014
YCB	3.5	28	2.9 (74)	4.9 (28)	6.196	<0.0001	2.9	28	3.0 (72)	2.9 (28)	−0.302	0.764
FNB	6.2	56	5.8 (46)	6.6 (56)	2.447	0.0162	6.3	71	5.6 (29)	6.6 (71)	2.681	0.0105
NFB	5.9	58	5.4 (44)	6.4 (58)	3.054	0.0032	5.8	69	6.0 (31)	5.7 (69)	−0.927	0.3568
STB	6.1	65	5.8 (37)	6.3 (65)	1.483	0.1434	6.1	69	5.9 (31)	6.2 (69)	0.681	0.4979
Bitterness												
ST	5.8	54	5.0 (48)	6.5 (54)	4.746	<0.0001	5.4	61	4.3 (39)	6.1 (61)	5.555	<0.0001
IT	5.0	37	4.7 (65)	5.7 (37)	3.529	0.0006	5.1	56	4.4 (44)	5.7 (56)	4.558	<0.0001
CC	5.2	49	4.5 (53)	6.0 (49)	4.075	<0.0001	5.0	54	3.9 (46)	5.9 (54)	6.141	<0.0001
NF	4.2	26	3.9 (76)	5.3 (26)	4.163	0.0001	4.3	40	3.8 (60)	5.0 (40)	3.33	0.0012
AS	5.5	39	5.0 (63)	6.2 (39)	3.738	0.0003	5.4	58	4.3 (42)	6.2 (58)	5.598	<0.0001
KN	2.7	14	2.5 (88)	4.1 (14)	3.613	0.0021	3.0	25	2.9 (75)	3.5 (25)	1.467	0.1513
SG	5.3	47	4.6 (55)	6.2 (47)	5.237	<0.0001	5.0	53	4.0 (47)	5.9 (53)	5.460	<0.0001
OCB	4.4	24	4.2 (78)	5.3 (24)	3.399	0.0015	4.2	33	3.6 (67)	5.5 (33)	6.555	<0.0001
YCB	2.6	6	2.5 (96)	4.0 (6)	2.771	0.0331	2.5	13	2.5 (87)	2.5 (13)	−0.062	0.9512
FNB	6.2	65	5.2 (37)	6.8 (65)	4.940	<0.0001	6.0	72	6.0 (28)	5.9 (72)	−0.048	0.9616
NFB	5.5	53	4.9 (49)	6.0(53)	3.510	0.0007	5.1	62	5.0 (38)	5.2 (62)	0.420	0.6755
STB	5.8	53	5.1 (49)	6.4 (53)	4.661	<0.0001	5.2	58	5.2 (42)	5.3 (58)	0.231	0.8178
Astringency												
ST	6.1	65	5.5 (37)	6.5 (65)	3.236	0.0019	5.3	66	4.9 (34)	5.5 (66)	1.289	0.2021
IT	5.3	56	4.6 (46)	5.9 (56)	4.146	<0.0001	5.4	68	5.0 (32)	5.6 (68)	1.837	0.0704
CC	5.1	50	4.2 (52)	6.0 (50)	6.017	<0.0001	5.0	52	3.9 (48)	6.0 (52)	6.083	<0.0001
NF	4.7	48	3.9 (54)	5.6 (48)	5.284	<0.0001	4.9	58	4.0 (42)	5.5 (58)	4.210	<0.0001
AS	6.0	69	5.1 (33)	6.5 (69)	4.495	<0.0001	5.7	60	4.2 (40)	6.7 (60)	7.975	<0.0001
KN	3.1	19	2.7 (83)	4.9 (19)	5.064	<0.0001	3.4	33	3.3 (67)	3.6 (33)	0.932	0.3546
SG	5.6	57	4.6 (45)	6.4 (57)	5.690	<0.0001	5.8	59	5.2 (41)	6.2 (59)	3.603	0.0005
OCB	4.8	28	4.7 (74)	5.2 (28)	1.593	0.1172	4.4	37	3.8 (63)	5.4 (37)	4.523	<0.0001
YCB	2.7	16	2.4 (86)	4.2 (16)	5.177	<0.0001	2.6	23	2.5 (77)	2.7 (23)	0.499	0.6201
FNB	6.4	76	5.3 (26)	6.8 (76)	4.143	0.0002	6.1	68	5.9 (32)	6.2 (68)	0.840	0.4049
NFB	5.7	59	5.0 (43)	6.2 (59)	3.853	0.0002	5.2	58	5.4 (42)	5.0 (58)	−0.880	0.3814
STB	6.2	64	5.5 (38)	6.7 (64)	3.363	0.0015	5.4	65	5.5 (35)	5.3 (65)	−0.380	0.7045
Aftertaste												
ST	6.3	65	5.9 (37)	6.5 (65)	2.138	0.0355	5.8	66	5.2 (34)	6.2 (66)	2.771	0.0072
IT	5.6	56	5.3 (46)	5.9 (56)	2.092	0.0394	5.6	68	5.3 (32)	5.7 (68)	1.377	0.1735
CC	5.6	50	5.1 (52)	6.1 (50)	3.338	0.0012	5.8	52	5.0 (48)	6.5 (52)	4.042	0.0001
NF	5.5	48	5.3 (54)	5.6 (48)	0.964	0.3372	5.1	58	4.5 (42)	5.6 (58)	3.178	0.0021
AS	6.2	69	5.6 (33)	6.6 (69)	3.249	0.002	5.9	60	5.2 (40)	6.4 (60)	3.325	0.0014
KN	3.9	19	3.9 (83)	4.3 (19)	1.081	0.2877	4.2	33	4.2 (67)	4.3 (33)	0.387	0.6995
SG	6.0	57	5.2 (45)	6.5 (57)	3.576	0.0006	5.8	59	5.2 (41)	6.2 (59)	3.630	0.0005
OCB	5.9	28	6.0 (74)	5.5 (28)	−1.519	0.1357	5.6	37	5.3 (63)	6.1 (37)	2.636	0.0103
YCB	3.5	16	3.4 (86)	4.2 (16)	1.803	0.0863	3.2	23	3.2 (77)	3.2 (23)	0.187	0.8526
FNB	6.6	76	5.8 (26)	6.9 (76)	2.545	0.0154	6.4	68	6.2 (32)	6.6 (68)	1.034	0.306
NFB	6.2	59	5.9 (43)	6.5 (59)	1.692	0.0949	5.7	58	5.6 (42)	5.9 (58)	0.837	0.4052
STB	6.2	64	5.7 (38)	6.5 (64)	2.234	0.0294	6.0	65	6.2 (35)	5.9 (65)	−0.828	0.4097

ST, Suntory Oolong tea; SG, Sangaria Oolong tea; CC, Coca-Cola Oolong tea; NF, Oriental Leaf Oolong tea; AS, Asahi Oolong tea; KN, Golden Flowers Oolong tea; IT, Ito En Oolong tea; OCB, OCOCO; YCB, YU-CHA; FNB, Tominaga; NFB, Oriental Leaf Black Oolong; STB, Suntory Black Oolong tea.

## Data Availability

The datasets presented in this article are not available because of ethical restrictions.
